# Supplementary material for: Light Exposure, Physical Activity, and Indigeneity Modulate Seasonal Variation in NR1D1 (REV-ERBα) Expression
Source: Biology (Basel). 2025 Feb 25;14(3):231. doi: 10.3390/biology14030231 (PMC11939400; doi:10.3390/biology14030231)
Supplement: Supplementary file 1 [file biology-14-00231-s001.zip › biology-3474282-supplementary.pdf]

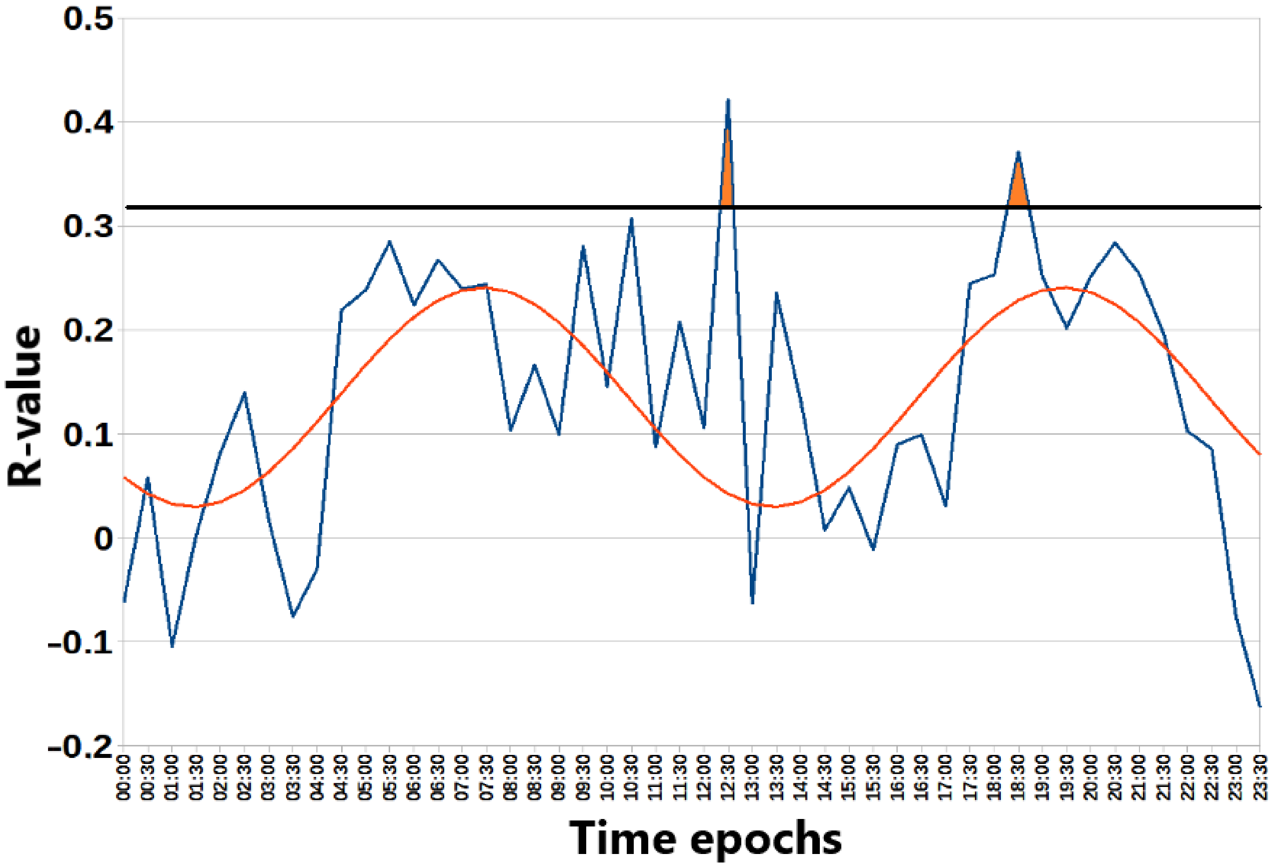

Supplementary Figure S1. Chart of r-values from a linear regression of NR1D1 (REV-ERBα) expression with physical activity (ordinate) in consecutive 30-min time epochs (abscissa). Horizontal black line corresponds to the threshold of significance after Benjamini–Hochberg's correction for multiple testing at 0.1. Blue curve: cosinor 12-hour model rejects zero-amplitude assumption of no rhythmicity; F=10.5; p=0.0002, approximation validates two peaks at 07:26 and at 19:26. Correlation of the higher physical activity with the higher NR1D1 (REV-ERBα) expression after correction for multiple testing is significant during time epochs shaded in orange.

Supplemental Table S1. Light exposure MESOR characteristics by season.

Season	Light Exposure (LE) Mean	SD	SE	95% Confidence Interval
WINTER SOLSTICE	23.68	13.92	1.84	19.98 ; 27.37
SPRING EQUINOX	74.01	46.88	5.86	62.30 ; 85.72
SUMMER SOLSTICE	213.67	234.66	42.15	127.60 ; 299.75
AUTUMN EQUINOX	40.97	20.19	3.04	34.83 ; 47.10

SD – Standard deviation, SE – standard error. Post-hoc Tukey Honestly Significant Test verify summer light Exposure MESOR is significantly higher than in any other season ($p<0.0001$), and winter light Exposure MESOR is significantly lower than in spring ($p<0.05$).

Supplemental Table S2. Light exposure phase characteristics by season.

Season	Light Exposure (LE) Mean	SD	SE	95% Confidence Interval
WINTER SOLSTICE	14:14	01:15	00:09	13:54 ; 14:34
SPRING EQUINOX	12:57	00:58	00:07	12:42 ; 13:11
SUMMER SOLSTICE	14:12	01:36	00:17	13:36 ; 14:48
AUTUMN EQUINOX	13:00	00:56	00:08	12:43 ; 13:17

Phases are significantly later during solstices vs. equinoxes, but highly similar in both solstices and equinoxes. Overall earliest / latest phases of light exposure are 10:20 and 18:08. SD – Standard deviation, SE – standard error.

Supplemental Table S3. No evidence for seasonal differences in MESOR of physical activity.

Season	Physical Activity MESOR (PIM) Mean	SD	SE	95% Confidence Interval
WINTER SOLSTICE	2421.07	598.03	79.21	2262.39 ; 2579.75
SPRING EQUINOX	2553.66	712.73	89.09	2375.62 ; 2731.69
SUMMER SOLSTICE	2449.26	728.28	130.80	2182.12 ; 2716.40
AUTUMN EQUINOX	2682.86	749.60	114.31	2452.17 ; 2913.55

PIM – Power Integrative Mode; SD – Standard deviation; SE – standard error.

Supplemental Table S4. Correlation matrix for associations between NR1D1 (REV-ERBα) expression with variables monitored by actigraphy**.**

Variable	Correlation coefficient
Physical Activity (Proportional Integration Mode, PIM)	
MESOR	**0.393148**
Amplitude	**0.273181**
Phase	-0.011691
Intra-daily variability (IV)	-0.033803
Inter-daily stability (IS)	0.157265
M10	**0.396042**
M10 Onset	0.031494
L5	0.140206
L5 Onset	0.049544
Relative Amplitude	0.068491
Wrist Temperature	
MESOR	0.121090
Amplitude	-0.183083
Phase	-0.165673
Light Exposure	
Light Exposure MESOR	**0.371175**
Light Exposure Amplitude	**0.350618**
Light Exposure phase	**-0.221600**
Blue Light Exposure MESOR	**0.360930**
Blue Light Exposure Amplitude	**0.338768**
Blue Light Exposure phase	-0.219735
Blue Light Exposure M10	**0.358061**
Blue Light Exposure M10 Onset	0.155940
Blue Light Exposure L5	0.214163
Blue Light Exposure L5 Onset	0.068686
Blue Light Exposure Relative Amplitude	0.159261
Blue Light Exposure Normalized Amplitude	0.033347
Sleep Characteristics	
Bed Time	0.044378
Wake Time	**0.274046**
Total Sleep	0.202415
Sleep Efficiency	-0.099363
Wake After Sleep Onset (WASO)	0.095276

Note that light phase did not cross midnight, ranging from 10:20 to 18:08 with narrow 95% confidence interval (13:21;13:43) around the mean 13:32.
